# Beyond Just Talking Strategy: Using Gaming Simulations to Catalyze Airline Managers’ Buy-in to Novel Strategies that Can Shape or Adapt to Profit Cyclicality

**DOI:** 10.1007/s11213-023-09650-2

**Published:** 2023-06-01

**Authors:** Paul A. Langley

**Affiliations:** Westminster Business School, University of Westminster, London, UK

**Keywords:** Airline cyclicality, Dynamic complexity, Gaming simulations, Gaming to learn, Management buy-in, Strategic decision-making

## Abstract

This empirical qualitative study explores the role of gaming simulations in catalyzing changes to organization-wide management’s perspectives on a novel strategy for aircraft orders and retirements. A large US airline developed the new strategy to tackle the pervasive problem of profit cyclicality, driving poor average profit performance across the cycle. Based on the dynamic model used to develop the strategy with senior management, a gaming simulation workshop was designed and delivered in groups of 20 to over 200 organization-wide managers. They tested various strategies for aircraft orders and retirements, under scenarios for market demand and conduct for competitors and regulators.

A qualitative methodology was used to capture the workshop participants’ perspectives on the efficacy of various capacity strategies, before, during and after the workshop. The findings are that managers experiment risk-free with innovations in strategies for capacity orders and retirements and they do indeed discover for themselves that there are counterintuitive alternatives that can achieve large and stable profitable growth. These strategies depend on competitors (role-played by workshops participants in the simulation) cooperating to create a win-win equilibrium. Performance far exceeds the industry benchmark profit cycle.

The contribution is the empirical evidence of the effectiveness of gaming simulations to catalyze managers’ shared beliefs and buy-in to a new strategy or business model. There are implications for practitioners in airlines and other sectors on the use of a gaming simulation workshop toolset, to help create such buy-in for an emerging strategy or business model. Protocols for best practice gaming simulation workshop design are discussed.

## Introduction

This research is based on consulting work with a major US airline which was struggling with a 44-year old pervasive challenge - how to manage profit and growth across the industry cycle. For much of the past 44 years, since deregulation in 1978, industry demand has grown positively every year, closely matched to GDP growth rates. Although demand growth rates do vary in a cyclical manner, profits have varied enormously across the same cycle. Booms are followed by busts, which are followed by more booms. The US airline senior management team explained their perspectives on the sector:

*It’s a Cyclical Industry - There is Nothing Much that We Can Do About it!*

*Whenever we thought we had figured out the top of the cycle, it came much later!*

But a number of industry observers have claimed recently that profitable growth is here to stay because the industry structure has changed, including more flexible capacity, lower fixed costs, and more sophisticated options for aircraft orders, leases and retirements (Boeing 2022; IATA 2022; McKinsey & Company 2022; Schäfer 2017).

**Fig. 1:**
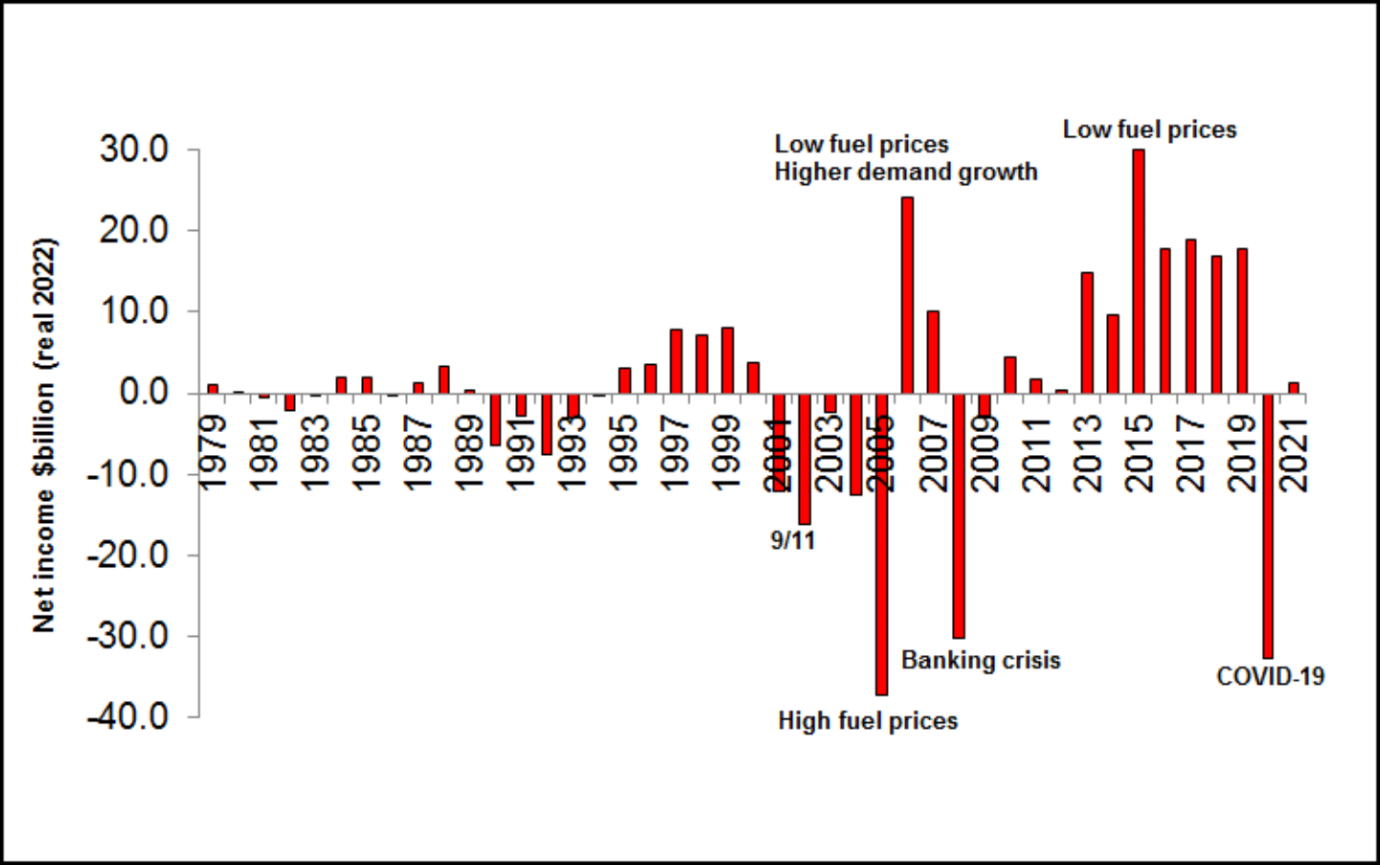
US airlines net income 1979–2021 (real 2022 US$ billion). Source: International Air Transport Association (IATA), Bureau of Transportation Statistics (BTS)

US airline industry profits have been positive for 11 years from 2010 to 2021, apart from 2020 with a discontinuity from COVID-19 (Fig. 1).

This is in complete contrast to the cyclic performance in the previous decade 2000–2009, with discontinuities in 2001 (9/11 terrorist attack) and 2008 (global financial crisis). Similarly for the cyclical 20 years 1979–1999. Wall Street apparently agreed. The airline industry’s historically low price to earnings ratio (around 10, compared to an S&P average of around 16) reflected investors’ concerns about the stability of earnings (Bloomberg 2022). In fact, over 43 years from 1979 to 2021, the average net income of US airlines is just $0.94b (real 2021), with a range from -$37b to +$30b. The shift in recent airline financial performance owes much to a globally recession-free cycle during the 2010–2021 decade. And the major global financial crisis at the end of the previous decade, together with the actions that carriers subsequently took, laid the groundwork for airlines’ improved profitability for the next ten years.

But not all airline executives have short memories. Some fear the cyclicality will soon return. Early-20th-century Spanish philosopher and writer George Santayana famously said, “Those who cannot remember the past are condemned to repeat it.” (Santayana 1905). The top questions from the client US airline CEO to the Board included:

1. How can we shape the industry to improve average performance across the cycle?
2. How can we adapt better to the cycle and improve our own performance?

In response the Board worked with the strategic initiatives team to develop a new business model and strategy related to fleet capacity – aircraft orders and retirements. This was a novel strategy which was radically different from the historical experience for this airline and indeed the whole industry. The strategy involved constant orders across the cycle and depended on cooperation from competitors (Friedman 1971; Axelrod 1981). Now the challenge was how to get buy-in from over 200 airline managers before any attempt to start implementation of the strategy (Hensher 2013). And to create a perspective shared by managers throughout the organization that the new strategy is the right one.

One natural option would have been to synthesize the rationale for the new strategy and brief all general managers accordingly, to kick start the change momentum in the organization (Neto and Barcellos 2022). The problem was that the rationale would appear to be counterintuitive to many. A different approach was adopted organization-wide based on gaming simulation workshops (Sterman 2010; Morecroft 2015). A competitive simulation game for 20 or so managers was designed, which enabled them to “experiment” with the new strategy for themselves. User-friendly gaming interfaces were linked to the dynamic modeling toolset which had been used to develop the strategy for the senior team. Manager participants role-played airline competitors.

This study builds on existing research on the impact of gaming simulations to help management teams, where multiple barriers to learning are found (Augier et al. 2018; Torres et al. 2017). Gaming simulations have been used variously, including helping to decide the best strategy or tactics in specific situations; convincing managers organization-wide that the new strategy is the right one; and learning how best to work with a new business model – by unfreezing, refreezing and then re-grooming.

This study evaluates the impact of these gaming simulation workshops on management learning and buy-in about the new strategy. A qualitative methodology was used to capture the workshop participants’ perspectives on the efficacy of various capacity strategies, before, during and after the workshop. Does the management buy-in involve concurrence, acceptance and willingness to actively support the new strategy? Three kinds of data collection were used: interviews, observation and documents (Vesa and Vaara 2014). The data from transcripts of participant interviews and questionnaires were analyzed using standard thematic qualitative coding techniques (Flick 2014).

The findings are that the managers experiment risk-free with innovations in strategies for capacity orders and retirements and do indeed discover for themselves that the alternatives, albeit somewhat counterintuitive, appear to work well. The new strategies shape the industry conduct or adapt better to the profit cycle. But they depend on competitors (who managers have role-played in the simulation) being cooperative and understanding capacity and price signals. We also find that a number of workshop protocols (e.g., overcoming “opaque boxes don’t sell”) are crucial to creating the right environment conducive to managers changing their entrenched perspectives. And further to just communicating the new strategy to organization-wide managers, the workshops generate recommendations for refinements to the senior strategy development team.

The paper continues with a “Theoretical Background” followed by a description of the “Methodology” used to collect reflections on learning from the gaming simulation workshop participants. Then there is a summary of the “Results” found and a “Discussion” of their significance. Finally the paper ends with a “Conclusion” and “Limitations of the study with suggestions for further research”.

## Theoretical Background

Three complementary themes from the transport and management fields are examined – modeling strategies to beat airline cyclicality; the challenges of dynamic complexity and counterintuitive outcomes; and management learning about dynamic complexity.

### Modeling Strategies to Beat Airline Cyclicality

A number of previous studies on airline industry profit cyclicality have focused on related issues. Skinner et al. (1999) discuss work undertaken at McKinsey & Company with airline industry clients to understand how to shape or adapt to the profit cycle. The main drivers of industry profit cycles are articulated as industry structural (e.g., manufacturing lead times that extend beyond the demand forecast horizon, combined with high fixed costs) and conduct factors (e.g., aggressive aircraft orders in the up-cycle) that result in persistent supply-demand imbalances.

Airlines place orders during periods of robust growth. Unfortunately, industry players cumulatively order more capacity than is necessary to meet demand growth. After a delivery delay for aircraft of two years or so, when they arrive core-demand growth is often not quite so robust as it was when the orders were placed. Airlines react to the over-capacity by cutting prices. Poor yields and low load factors lead to poor profit performance. Airlines react by retiring aircraft, pulling out of markets, and canceling remaining orders. Bankruptcies are common (Cronrath 2017; Samunderu 2019).

A business dynamics simulation model was developed, validated, tested and executed to test various capacity orders and retirements strategies, under a variety of competitor and market scenarios. The system (business) dynamics methodology is ideally suited to studying cyclicality, because it models structure and conduct that generates the cyclical performance. Behavioral decision rules are modeled to capture stakeholder conduct (airlines, consumers, manufacturers, regulators).

Pierson and Sterman (2013) cite earlier studies with system dynamics models (e.g., Liehr 2001) and add to their model endogenous feedback considering price setting, wages, and air travel demand. Insights are that aggressive use of yield management - varying prices to ensure high load factors (capacity utilization) - may have the unintended effect of increasing earnings variance. More recent work adds dynamic complexity to an understanding of niche contexts, including short-haul vs. long-haul business models (Urban and Hornung 2021); Covid-19 impact (Renold et al. 2023); low costs vs. full service business models (Maung et al. 2022) and airline capacity discipline (Hazel 2018). Various modeling approaches for capacity planning in transportation systems are compared (Cunha et al. 2022). A system dynamics model solution helped to understand the conduct of airline industry stakeholders, which are dynamic over time (Sgouridis et al. 2011). The stakeholders’ reaction to external changes (e.g., economic externalities and fuel prices) triggers changes to internal conduct (e.g., yield management, fleet orders and retirements).

### Challenges of Dynamic Complexity and Counterintuitive Outcomes

Empirical results from managerial labs and real-world case studies show that across the different complex dynamics and sector contexts, managers have difficulties in understanding very basic stocks and in-out flows (Gary et al. 2008). They also underestimate the role of time delays between decisions and outcomes as these stocks accumulate over time. Hence performance outcomes will often demonstrate overshoot, collapse and cyclicality.

These dynamic complex environments are often difficult to manage well, because they appear to be deceptively easy situations to cope with (*misperceptions of feedback*, Sterman 1989). However, it’s never as easy as it first appears (Bartunek and Moch 1987). The unintended consequences of actions often counterbalance (or indeed countermand) many managerial initiatives. Also, many managers under-invest the time to understand the second, third and fourth order effects of their decisions. These “cognitive limitations” about dynamic complexity can be overcome with the help of dynamic modeling and simulation tools (Kazakov and Kunc 2016).

Business model success is linked to the mastery of dynamic complexity, i.e., developing perceptions that can identify levers which drive self-reinforcing feedback loops. An example is that although a business model might lead to impressive financial and sustainability performance under most market demand and regulatory scenarios, it may show poor resilience to competitive threats. Moreover, the entrance of new competitors with a larger network size turns the business model’s virtuous cycle into a vicious cycle and leads to reinforcing losses (Täuscher 2018). The recent digitization trends in rapidly evolving technologies, consumer preferences and new business models have significantly increased the speed and magnitude of change to generate sector discontinuities and multiple challenges for organizations to respond effectively (Langley and Rieple 2021).

Dynamic complexity is pervasive across many sectors and has archetypal performance characteristics, including:

**Cyclical performance** is a common source of management frustration, in which short-term actions often frustrate long-term results. For example, increasing demand and sustained profitable performance may trigger a new investment cycle. By the time the new capacity comes online, demand has started to fall again resulting in profit pressure and poor performance (Skinner et al. 1999).

**Pricing decisions** may affect sales volume in the short term, but also incur unintended side effects such as competitor response, capacity shortage, quality problems and revised expectations by the market that make it difficult to restore higher prices later on (Keith et al. 2017).

**Boom and bust** occurs when the market’s enthusiastic take-up of a new product is followed by a rapid drop in sales. Companies consistently get into trouble in these rapid growth markets. Frequently they grow too fast, overshoot when the market saturates, then get into price wars and suffer huge losses due to low prices and excess capacity (Gary et al. 2008).

**Growth slow-down**, with the advent of competitors together with other constraints undermining continued growth, strategies that were effective during the initial growth period are now no longer effective. Which leading indicators could have predicted the slow down? And which management strategies will work now? (Langley et al. 2000).

**Product portfolio management**, where product decisions impact the performance of other products due to cannibalization and resource diffusion. (Thrane et al. 2010).

### Management Learning About Dynamic Complexity

Winning in today’s complex world requires senior managers to explore new strategies together, in order to prepare creative responses to rapid change (Torres et al. 2017). Discussions about strategy are common and useful: “Under what conditions would this likely fail?”, or “What if the competition matches our move?” Nevertheless, dialogue may not suffice to realize change, for while managers might agree how they ought to respond under different scenarios, competitive or market pressures typically force managers to fall back on comfortable responses based on earlier experiences - “old habits die hard.” To readily respond to new opportunities, it is necessary to internalize strategic insights, as if one had actually *experienced* them for oneself. But business experience normally takes years to accumulate, so how can companies benefit from hands-on learning when risky actions may have irreversible consequences, months or years after decisions have been made?

Senior managers in organizations facing a major market change, where fundamental strategic choices need to be made, also need to build a shared understanding of the market for success (Van der Heijden 2011). This has three components. First, a shared understanding is required of the threats, challenges and opportunities that the new environment provides. Second, the creative energies of the entire management team are needed to develop innovative solutions. Finally, a need for a management team with an agile mindset, ready to react as the strategy unfolds. Building a shared vision is challenging when the firm is faced with rapidly changing industry structures, market discontinuities and other uncertainties, including technological change, new ways to compete, new entry competition and regulatory change.

To address this challenge, organizations are using gaming simulation workshops, which combine dynamic simulation models with user-friendly gaming interfaces. Augier et al. (2018) provide a recent review of the field. They introduce “doing” into strategy discussions in a fast, effective, and risk-free way. They act as a catalyst to help managers focus, reflect, and hence change their “mental models” and subsequently their strategic decision-making behavior.

Thus microworlds are powerful devices for communicating and internalizing strategy across an entire senior management team, dramatically accelerating learning about a shared vision of the firm’s future (Gary et al. 2012). The “gaming is learning” theme examines how managers explore strategic issues together in a risk-free environment, to gain wisdom on difficult strategic problems (De Geus 1988; Kark 2011; Waller et al. 2014). Higher order learning is escaping from an entrenched perception and implementing a mind-set different from the old one (Espedal 2008).

The gaming simulation that we will focus on in this paper is based on the system (business) dynamics field which develops continuous simulation models of economic, social and organization structures, combined with behavioral decision-making rules driving managerial conduct (Sterman 2010). The gaming interfaces attached to the simulation models are typically called *Microworlds* (Morecroft 1988) based on earlier ideas from Papert (1980) and Schön (1983). Also termed *Management Flight Simulators (*Sterman 2010*).*

Gaming simulations (GS) can help management teams in roles of both strategy formulation and implementation. Table 1 explains six different such roles.

**Table 1:** Gaming simulations (GS) role in impacting management learning about strategy

Role of GS	Explanation of Impact on Management Learning
Shock-test new strategies	GS allow strategies to be tested for robustness to various market and competitor scenarios. Managers can explore alternative, unfamiliar futures that are characterized by much uncertainty, learning to recognize signals from the competitive or market environment.
Improved cross-functional team collaboration	GS offer an opportunity for managers to change roles, and look at things from a new perspective. For example, a marketing manager may gain new understanding of the production manager’s position when trying to meet an aggressive production plan.
Understanding the competitor	GS allow managers to “see” a rival’s perspective of the market dynamics. For example, a competitor may be laden with debt and under great pressure to generate positive cash-flows. Alternatively, a competitor CEO may have a personal goal to become the market share leader “at whatever the cost”.
Dealing with tradeoffs (balanced scorecard)	Real business situations involve trading off different objectives, such as profit and long-term growth. GS can reflect these tradeoffs and show performance on different dimensions for comparison. During GS workshops, participants find that actual performance is a less important outcome than the awareness of tradeoffs that resulted. Comparing the different dimensions of performance force players to articulate their strategies in terms of multiple objectives, elevating the discussion to a higher level.
Explain performance	Well-designed GS allow the participants to track performance and explain retrospectively why they did well or poorly, forcing them to articulate their understanding of how strategy and tactics drive results. In dynamically complex situations, this capability is invaluable for learning.
Building a shared understanding	GS create a common focal point (often termed a “transitional object”) that is at the centre of a discussion about the business. They provide a vehicle to stimulate discussion about the environment in which each participant plays a part, thus forcing managers to state explicitly their views about “how things work”. This often creates noticeable changes in managers’ understanding of how the business works, as they try to reconcile their own views with those of their colleagues during GS playing and debriefing.

This last role of “building a shared understanding” or “buy-in” has few previous studies, examining how gaming simulation workshops can help communicate new strategies or business models to a wider group of managers, across the organization. Hence this study’s research questions are designed to fill a gap in knowledge, related to understanding the role of these gaming simulation workshops to:

- overcome the barriers to learning about dynamic complexity that drives historic airline poor average profit performance across the cycle.
- understand a new fleet capacity ordering and retirements strategy, despite it being somewhat counterintuitive.
- catalyze managers’ buy-in and shared belief in the novel strategy.

The specific research questions are how do managers’ perspectives on the efficacy of various capacity strategies change before, during and after the workshop? And how do manager participants discover that there are counterintuitive alternatives that can achieve large and stable profitable growth? Finally, does the management buy-in involve concurrence, acceptance and willingness to actively support the new strategy?

To answer these research questions, the next section describes the methodology used to collect the qualitative data from the gaming simulation workshops.

## Methodology

### Dynamic Simulation Model Development and Testing

This research study is based on the use of a proprietary business dynamic model, developed for the US airline client, as part of a strategy development process. The US airline’s proposed strategies are confidential, but we can discuss them at a generic level to understand the insights that the gaming workshops were designed to communicate to organization-wide managers. An earlier version of the simulation model is documented in Skinner at al. (1999).

Later versions are not published, but the model structure and the capacity ordering and retirement strategies are now described in outline here, so that the reader can better understand the challenges of buy-in which the airline organization’s managers faced.

**Fig. 2:**
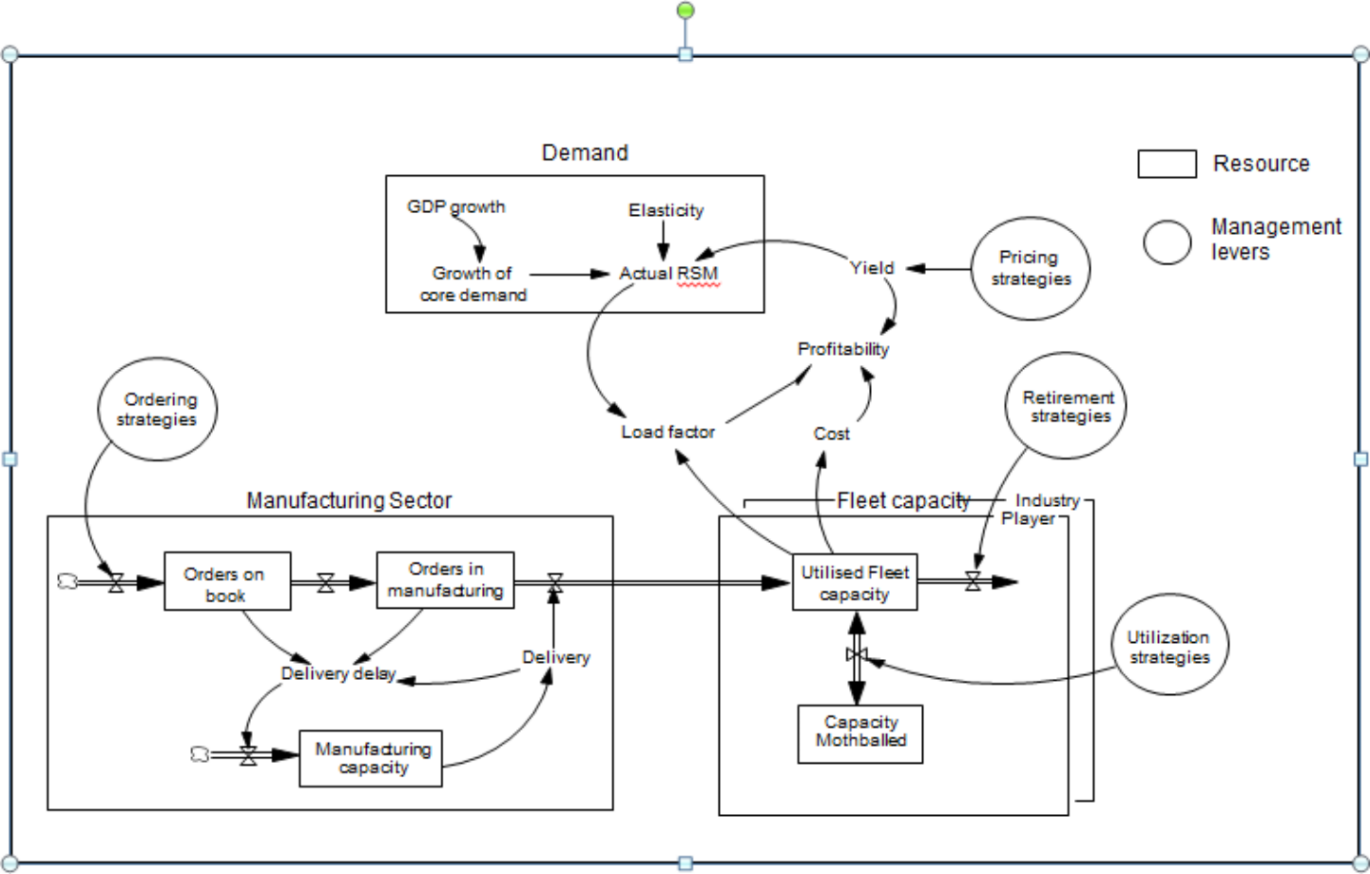
Block diagram feedback structure of the airline dynamic model (simplified from the client model) Note: RSM = Revenue Seat Miles

The dynamic simulation model of the airline industry represents the resources and capabilities that are owned by industry stakeholders (airlines, manufacturers, leasing companies). It models how these resources grow and deplete over time, driven by management levers (e.g., fleet capacity ordering and retirements, short-term leasing, pricing). The model also represents the behavioral decision rules that change the management levers over time. These rules represent realistic management decision-making. The detailed description of model validation and testing, with historic data and airline experts, is beyond the scope of this paper, but the best-practice processes are documented in detail elsewhere (Sterman 2010). Figure 2 shows the simplified feedback structure of the dynamic model, including airline strategies (behavioral decision rules) for pricing, capacity orders, retirements and utilization.

**Fig. 3:**
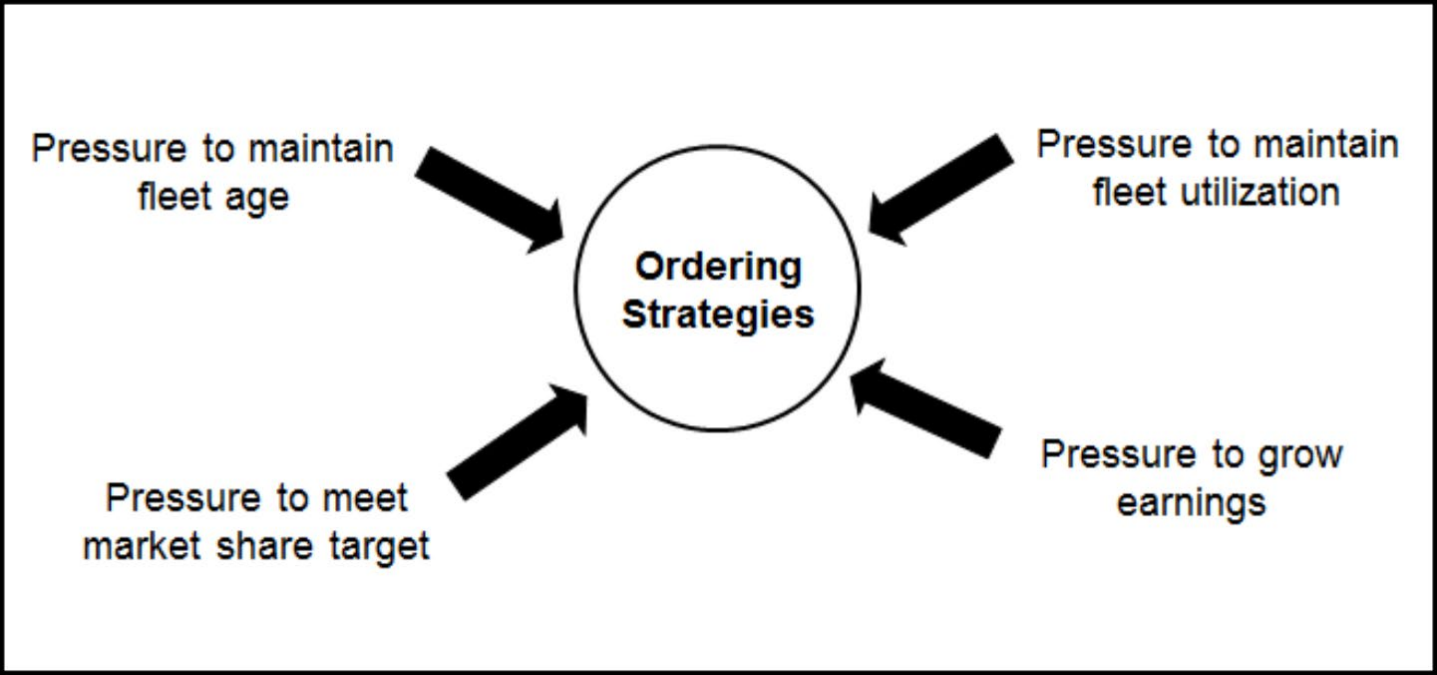
Pressures influencing capacity ordering strategies

Each strategy is driven by multiple “pressures”, either endogenous to the dynamic model or exogenous. For example, the capacity ordering strategy illustrated in Fig. 3 shows four such pressures, related to the age of the fleet, market share targets, earnings growth expectations and fleet utilization targets. The behavioral decision rules are designed following interviews with airline managers responsible for the particular strategy and calibrated using historical data.

Wholesale changes in industry structure and conduct can significantly improve performance across the cycle, driven by a distrust of industry wisdom, a flexible capacity structure, and a cycle-driven approach to investments. There *are* specific strategies that can help airlines improve financial performance across the cycle, by shaping or adapting to the profit cycle. For example one strategy, the *Flexible Reactor*, nearly doubled cumulative return on invested capital over our simulated period. Although its performance is still highly cyclical, its peaks are well above those associated with the other strategies, and its troughs rarely below them.

**Fig. 4:**
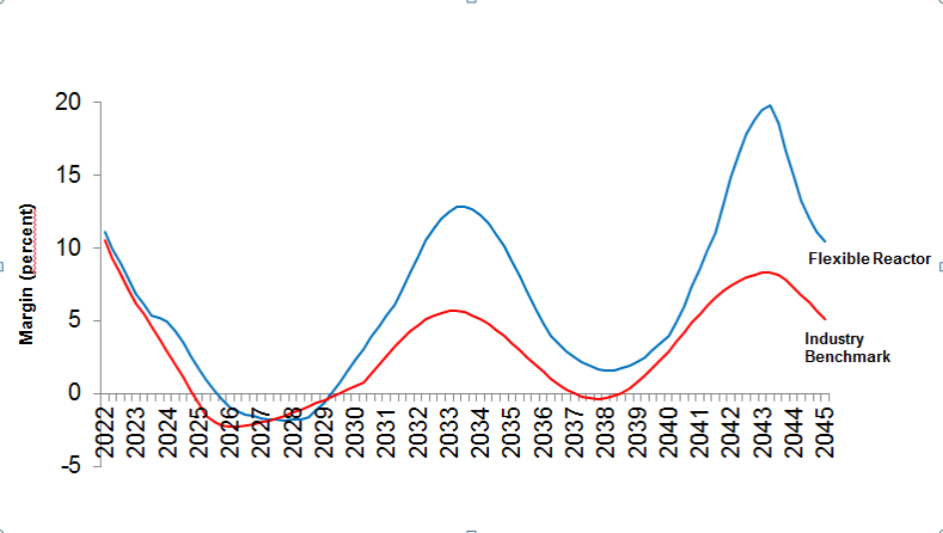
Airline operating margin (percent) from 2022–2045. Source: dynamic simulation model

The improved ability to manage growth across the cycle translates directly into improved investor confidence and higher price to earnings (P/E) ratios. A major airline that improves its P/E to the average of the Standard & Poor’s (S&P) 500 could increase its market capitalization by as much as 250%. By placing large long term steady orders across the cycle, and adjusting to short term market conditions (measured by incremental changes in revenue growth) by quick-response retirements and reductions in utilization. Retirements are volatile, with high sensitivity to market conditions, while orders are sensitive to only to long run growth trends. During high growth, the carrier simply stops retiring. Figure 4 shows the profit performance over 43 years for the flexible reactor vs. an industry benchmark. This benchmark is a “business as usual” (BAU) strategy, where capacity ordering and retirements follow historic custom and practice. This helps to validate the gaming simulation model with the workshop participants, because their expectations of familiar financial performance outcomes are met. Other strategies are possible with different performance outcomes, but are not documented in this paper.

But convincing managers throughout the organization of these counterintuitive findings was perceived by senior management as a formidable challenge. Why? It is conceptually difficult to accept that the airline competitors (and suppliers) would execute the discipline needed to cooperate with us, rather than out-compete us. More likely they would respond by investing aggressively in the up-cycle to steal market share (Hollander and Prashker 2006).

The proposed solution to gain organization-wide managers’ buy-in was to use a generic version of the airline dynamic simulation model as a basis for a gaming simulation workshop, which would be rolled out to 200 managers (in groups of 20) across the organization. This generic version avoided the detailed complexity of the clients’ own airline, hence the squabbling and “nit-picking” about errors in the detail. Instead the gaming simulation presented a 20% player in a near-oligopoly of five large market players (Bortolomiol et al. 2022). Hence very similar to the US airline industry structure post 1978 deregulation. And importantly, the dynamic complexity of the generic industry with five players was very similar to the real US airline industry.

The gaming simulation allowed workshop participants to role-play airline industry competitors (each with a 20% market share). Participant role-players could be replaced by model algorithms if required. For example, an airline competitor’s conduct on pricing, capacity and marketing decisions could be automated according to a pre-designed scenario.

### Data Collection from Management Gaming Simulation Workshops

A workshop based on a gaming simulation was designed to help the airline managers fully understand the industry dynamics. Then to think about developing fleet ordering and retirement strategies designed to mitigate and perhaps even take advantage of, the effects of business cycles. Finally, there would hopefully be a full or partial buy-in to the airline’s proposed new strategy to deal with future profit cycles. Does the buy-in involve concurrence, acceptance and willingness to actively support the new strategy (Hensher 2013)?

Teams of company managers played against competitors (either model-driven or client role-playing), in which they tried to create and capture as much value as possible. The game then catalyzed a process of well-structured discussion about the strategic choices and possible outcomes. Yearly decisions involved ordering and retiring fleet capacity (aircraft). Reports and graphs included operating and financial data. This gaming simulation was implemented at a very high level of aggregation but was still very effective as a learning tool.

Gaming simulation workshops were organized as half-day events for 20 participants, for a total of over 200 managers across the airline organization, over six weeks. Typically cross-functional teams were allocated to a workshop, which would involve a mix of plenary briefings and debriefings, game play, and feedback and reflection from each team. This study’s author was a workshop supporter and not the lead facilitator, which helped to avoid researcher bias influencing participant outcomes (Jarzabkowski et al. 2015). Participants were given little direction in choosing the capacity and retirements strategies – they were left to experiment as they wished, with an objective to maximize returns over 20 or so simulated years. Data for this research was collected from five workshops out of the total 20, spread over the six week timescale.

Our research methodology is qualitative, employing three kinds of data collection: interviews, observation and documents (Vesa and Vaara 2014). The data from transcripts of participant interviews and questionnaires were analyzed using standard thematic qualitative coding techniques (Flick 2014). This method was deemed appropriate as it can highlight similarities and differences across the data set and can generate unanticipated insights (Braun and Clarke 2006). The coding was based deductively on prior literature, and inductively on new insights emerging from the data, in a retroductive stance. To support data integrity, a synthesis of the main workshop learning was shared with a senior client present at the workshop, with a follow-up discussion to resolve inconsistencies.

Our workshops incorporated best-practice aspects of gaming simulation workshop design (Rumore et al. 2016; Augier et al. 2018; Torres et al. 2017). Table 2 shows the elements of best-practice gaming simulation workshop design together with the rationale for the adoption of each element.

**Table 2:** Elements of gaming simulation workshop design and the rationale for adoption

Element of workshop design	Rationale
Senior management is present	The presence of a senior member of the management team, at least at the beginning of the workshop, if not during the entire game, enhances involvement and buy-in to the learning process by managers.
Participants accept the gaming simulation as “fit for purpose”	So that participants concentrate on engagement with the gaming simulation, rather than criticizing it as inaccurate or not adequately validated. This important credibility is best achieved by explaining some structural elements of the simulation model. In this research there was additional credibility gained from using a gaming simulation model which was a generic version of the more detailed client model that had been used for strategy development with a senior team.
Learning is iterative	In successful workshops, participants play two or more times, thus reflecting on their performance and learning how to improve in each round. The desired outcome of faith in their ability to control their outcome is thus achieved.
Debriefing discussion follows each round	These are arguably the critical points in the workshop when key learning takes place. A discussion of workshop participants’ capacity ordering and retirement strategies and their intended versus actual performance outcomes is important to consolidate the learning from each gaming round. Furthermore, workshop competitors’ reactions to capacity signaling are crucial in this case, to assess participants’ learning to cooperate rather than compete.
Competition exists between teams	Measurable gaming simulation results (e.g., profit or shareholder value) can be compared between different teams at the end of the game. This stimulates more commitment to results among players, and more creative playing, as they try to beat their colleagues.
There is similarity between the game and the real world	Well-designed games use company terminology and replicate the appearance of company reports, thus increasing comfort and enhancing credibility among players. Multimedia materials (TV, newspaper and social media stories) add much realism here.
The gaming simulation is part of a larger strategic initiative	The workshop is not an end in itself, but is part of a larger purpose, such as a major shift is strategy. This enhances the importance and “sense of event” around the gaming simulation, and also focuses the discussion on larger objectives.
Duration of the gaming simulation introduces short-term pressures	Even when there is a prior discussion about long-term goals, well-designed gaming simulations cause people to react to short-term pressures, thus forgetting about the long-term and focusing instead on immediate fixes, such as price cuts or unwise capacity expansion. The gaming simulation must be rich enough and played long enough to allow such pressures to rise, introducing a rich basis for discussion later.
Players log their decision logic	During the game, players must log the reasons for their decisions. This increases the pressure to formulate an up-front strategy, and avoids “post-hoc” rationalization that can undermine the learning during debriefing.

## Results

Of the 200 or so airline managers who participated in the half-day workshops, many started out with similar beliefs. A mix of disbelief in, and disenchantment with, “yet another” new approach to dealing with profit cycles. Such approaches (“new business models”; “innovative strategies”) arrived annually as part of planning reviews.

*Profits have always gone up and down in this industry - there is nothing much that we can do about it!*

Beating airline cyclicality to improve average profit performance across the cycle – reduce losses in the bad years and improve profits in the good years – was seen as a wicked problem with no solution.

*Only the Big Players Could Really Influence the Cycle but They Ride Through it Anyway!*

*We Spend too Much Time Thinking About this Issue!*

One strategic approach was to manage flexible capacity, to meet uncertain demand in the cycle. For example, players experimented with a “Flexible Reactor” strategy and nearly doubled cumulative return on invested capital over 20 years. By placing large long-term steady orders, keeping baseline retirements high and ignoring short-term industry demand forecasts, they were able to maximize the peaks of the profit cycle, while minimizing the damage of the troughs. The “Flexible Reactor” strategy was not a new idea – it had been tabled numerous times for discussion at senior levels, but always quickly dismissed as impossible to implement.

*Competitors would take advantage of the situation and adapt their conduct to favor retaliatory ordering, thus leading to chronic overcapacity.*

In the workshop, competitors played by client senior managers were able to mirror the client strategy of constant orders across the cycle and created superior performance for all players in the industry. The discussion moved towards a new insight – all the major competitors in the oligopolistic airline industry could benefit from adopting the same strategy. The airline should think further about capacity (and price) signaling to achieve a cooperative equilibrium. Playing the role of competitors in the gaming simulation showed airline managers how this could be done.

*It’s good to see how constant orders across the cycle might play out. We’ve never had the courage to try that for real.*

*If you know where you are in the cycle then you can come up with a killer strategy!*

Managers do not go back to their desks after a single workshop with a “we have now seen the light” mindset to implement a change program without delay. However the opportunity to experiment “risk-free” with different approaches to managing fleet capacity generates at least an acceptance that the idea merits further thought.

*Our next steps are to articulate a set of proactive, no regrets moves that the gaming simulation workshop had identified as likely winners, regardless of how the market played out.*

*…and launch a focused, competitive intelligence effort in the coming months to actively manage a contingent strategy based on the remaining key uncertainties*.

The main dynamic complexity was the cyclicality in demand (driven by GDP cycles) is amplified many times to generate profit cycles and poor average profit performance across the cycles. The breakthrough in management learning was that industry players do much themselves to create the cycle, rather than demand externalities. Cyclical profit performance can be flattened by cooperative capacity conduct by all players, for example making constant capacity orders across the cycle. The strategic insights were enriched by shifting to thinking about creating a cooperative equilibrium between industry players – *how to make this happen*, rather than *this could never happen*.

*I didn’t realize how much the industry has only itself to blame for cyclicality.*

*One thing that our own fleet planning system is not doing currently is giving the big picture!*

Client managers said they found the simulation “amazingly realistic” (which was also reflected by the level of competitive intensity the players demonstrated). Interestingly, when asked in debriefing sessions to reflect on what they had learned in the game, the “insights” they had gained were exactly those intended, and were not rejected as “obvious”, or as specific to one business. For instance, the lesson that competitors must cooperate was clearly understood in a much more vivid way than when simply “stated”.

The workshop was also useful to help managers reflect on both their personal style of management (innovators innovated, the tame did not do anything radical and stagnated, “gung-ho” action types concentrated all their firepower on one strategy, but did not manage to choose the right one).

However, the “Flexible Reactor” strategy presumes a high degree of flexibility that few airlines currently possess.

*We could never do this in practice – the quick-response retirements and reductions in utilization would be nearly impossible to staff!*

The purpose of this exercise is to “learn” about the impact of different scenarios on future performance. The fact that this strategy outperforms the others is not terribly surprising. What is interesting, however, is by how much.

One caveat – there were concerns from a few participants about playing a 20% share generic airline, rather than the client airline.

*It’s a pity we can’t manage our own airline using this tool – there are many nuances here that we are missing!*

This led to discussions about a future gaming simulation workshop design with the detailed client dynamic model which had been used to build the client’s airline strategy, instead of the simplified generic dynamic model. And further how these “small” generic dynamic models could better fit with the “large” fleet planning tools.

## Discussion

The research questions are how do managers’ perspectives on the efficacy of various capacity strategies change before, during and after the gaming simulation workshop? And how do manager participants discover that there are counterintuitive alternatives that can achieve large and stable profitable growth? Finally, does the management buy-in involve concurrence, acceptance and willingness to actively support the new strategy?

The main finding from the participant observations and the verbal and written feedback was that managers’ perspectives changed about the new and somewhat counterintuitive strategy to deal with potential future profit cyclicality. They accepted the efficacy of constant orders across the cycle, combined with quick-response retirements and reductions in utilization. They understood the difficulties with implementing this strategy without a full cooperation of other industry players, which in turn might just be possible to achieve in a near industry oligopoly.

The US airline managers were able to experiment with constant aircraft orders across the business cycle, a strategy previously dismissed as too costly and had assumed that competitors would respond by investing aggressively in the up-cycle to steal market share. Managers’ perspectives changed on the likely success of constant orders (and flexible retirements) as they successfully executed such a strategy, including signaling to competitors, in the gaming simulation. The enriched strategic conversations discussed tentatively the possibilities for such a strategy to beat the cyclicality dynamics. What if the competition accepted the bait to cooperate with matched investments? Was a win-win in a cooperative equilibrium now possible?

The gaming simulation enabled managers to stress-test a new strategy or business model, i.e., strategies were tested for robustness to various market and competitor scenarios and trying to “shoot holes” in the robustness of the strategy. This follows the “gaming is learning” theme (De Geus 1988; Kark 2011; Waller et al. 2014) where managers explore together and gain wisdom in a risk free environment. It can include experimenting with approaches currently thought to be too risky to action for real. This management learning can be explained by a form of higher order learning, escaping an entrenched perception and implementing a mind-set different from the old one (Espedal 2008). Significant “deep” learning takes place when barriers to learning and managers’ defensive routines are overcome, facilitated by best practice workshop design elements explained in Table 2 in the Methodology Sect. 3.

And further to just communicating the new strategy to organization-wide managers, the workshops generate recommendations for refinements to the senior strategy development team. Existing beliefs were challenged and there is some evidence that changes to elements of the strategy were under consideration following the workshops. Certainly the next few weeks and months involved further strategic analyses and reconsideration of no-regrets next steps.

Managers perceived that the new strategy or business model was more robust than they had first thought, to competitor conduct or other market discontinuities or drivers of performance. This research makes an important contribution to knowledge about the role of gaming simulations to communicate a new strategy to organization-wide management and achieve their buy-in (Hensher 2013). Applicable to industries beyond airlines because complex dynamics are pervasive and can result in counter-intuitive outcomes across many organizational settings.

## Conclusions

Few previous studies have examined the role and efficacy of gaming simulation workshops as a part of strategy communication. In this research, prior to the workshops there was an iterative process of strategy development using a detailed dynamic model engaging a senior management team. Then we studied the communication of new strategic insights and the catalysis of shared beliefs and buy-in, through the gaming simulation workshops with organization-wide managers. The gaming simulation was based on a simplified generic model compared to the more detailed client model used to develop the airline strategy. Finally, there was further strategy refinement through feedback from the workshops to the strategy development team - a learning and improvement feedback process.

But what can be said about the credibility of the gaming simulations with managers? Why should they trust the simulated consequences of decisions on outcomes as being “fit for purpose”? The use of the “Industry Benchmark” case, with business-as-usual (BAU) capacity ordering and retirements strategies, generated expected cyclical financial performance, which managers had been familiar with historically. There was additional credibility gained from using a version of the simulation model used by the senior strategy development team. This version was a simplified version of the strategy development model, using generic players rather than the details of the client airline.

There are implications for practitioners in airlines and other sectors on the use of a gaming simulation workshop toolset, to help create such buy-in for an emerging strategy or business model.

### Limitations and Further Research

This research study collected data from managers in one US airline, participating in gaming simulation workshops. We would suggest that the findings might be extended to all US airlines, and indeed all global airlines, but we accept that such generalization may be hard to justify.

The workshops are certainly more risk-free compared to the real world. The consequences of failure – poor profit performance, or even worse a bankruptcy - are little more than an embarrassment. So some workshop participants may behave much less risk averse than they would in the real world, thus changing the gaming simulation outcomes. Strategic decisions driving performance outcomes could be “workshop biased” reflecting the relatively risk-free environment. Some of the gaming workshop protocols outlined in the methodology section helped to mitigate such undesirable behavior.

The issue of using a gaming simulation based on a simplified generic model (as we did in this study) vs. the detailed client model used to develop the airline strategy needs further research. In this study, the client had concerns over the managers’ “nit-picking” on irrelevant details that might occur if the detailed client airline model was used.

There is no attempt to assess the efficacy of gaming simulations vs. alternative strategy workshop designs, e.g., scenario planning and discussions. The literature has previously studied various approaches to generating strategy conversations in workshops (e.g., Healey et al. 2015; Seidl and Guérard 2015).

A quite different theme of research could study the impact of strategy workshops, with or without gaming simulations, on organizational performance. What is the cost-benefit of development and implementation? Do managers leave these workshops with changed mindsets and strategy perspectives, or do they continue with their entrenched views? These are challenging questions which would likely need a longitudinal study over an extended time period, potentially with multiple organizations.

## Data Availability

Not applicable.
